# DNMT1-dependent suppression of microRNA424 regulates tumor progression in human bladder cancer

**DOI:** 10.18632/oncotarget.4431

**Published:** 2015-06-10

**Authors:** Chun-Te Wu, Wei-Yu Lin, Ying-Hsu Chang, Paul-Yang Lin, Wen-Cheng Chen, Miao-Fen Chen

**Affiliations:** ^1^ Department of Urology, Chang Gung Memorial Hospital at Keelung, Keelung, Taiwan; ^2^ Chang Gung University, College of Medicine, Taoyuan City, Taiwan; ^3^ Department of Urology, Chang Gung Memorial Hospital at Chiayi, Puzi City, Taiwan; ^4^ Department of Urology, Chang Gung Memorial Hospital at Linko, Taoyuan City, Taiwan; ^5^ Department of Pathology, Chang Gung Memorial Hospital at Chiayi, Puzi City, Taiwan; ^6^ Department of Radiation Oncology, Chang Gung Memorial Hospital at Chiayi, Puzi City, Taiwan

**Keywords:** bladder cancer, miR424, DNMT1, EGFR

## Abstract

The aim of this study was to examine the role of miRNAs regulation by DNMT1 and its underlying mechanisms in bladder cancer. The choice of target miRNAs was based on the analysis of a TaqMan MicroRNA Panel assay. The role of target miRNA in tumor behavior and the related signaling pathways were assessed using the human bladder cancer cell lines. We also evaluated the predictive power of the target miRNA and its link to DNMT1 from 124 clinical bladder cancer specimens. Our results revealed that the miR-424 level is significantly increased when blocking DNMT1 in bladder cancer cells. From the clinical specimen analysis, the staining of miR-424 was inversely correlated with DNMT1 immunoreactivity. The lack of miR-424 expression was significantly linked to aggressive tumor growth, advanced clinical stage and poor prognosis in bladder cancer. Increased miR-424 suppressed the tumor growth rate and invasion ability determined *in vitro* and *in vivo*. Furthermore, the EGFR pathway plays a role in the transmission of the miR-424 signal that regulates cell growth and the epithelial-to-mesenchymal transition. These results highlight a potential role for miR-424 as a molecular predictor and therapeutic target in bladder cancer.

## INTRODUCTION

Bladder cancer is a significant public health issue worldwide and manifests itself in two distinct forms with different clinical and biological behaviors. Approximately 70% of patients presented with non-muscle-invasive tumors with good prognosis, and the remaining 30% with muscle-invasive tumors have a poor five-year survival rate [1, 2]. Although standard pathological features can be used for treatment decision-making, improving the ability to predict which patients will suffer disease recurrence and progression remains an important issue.

MicroRNAs (miRNAs) are single-strand RNA molecules with a length of 20-23 nucleotides that post-transcriptionally control gene expression [3]. Many miRNAs have oncogenic or tumor-suppressive actions [4]. A growing body of evidence suggests that miRNAs contribute to bladder cancer development, progression and metastasis [5, 6]. Thus, identification of aberrant miRNA expression and oncogenic molecular targets of miRNAs is important for potential biomarkers and the development of novel therapeutics for bladder cancer. Recent studies have shown that the silencing of several miRNAs in human cancers is tightly linked to epigenetic mechanisms, including DNA methylation [5, 7, 8]. DNA methylation is typically mediated by DNA methyltransferases (DNMTs), and aberrant DNMT expression facilitates malignant transformation, including urologic cancer [9, 10]. DNMT1 was reported to be over-expressed in several tumor types [11, 12], and play an important role in the prognosis of bladder cancer [13, 14]. It was recently shown that the silencing of miRNA expression in bladder cancer is associated with DNA methylation [15]. Thus, alterations in miRNAs expression may represent a mechanism responsible for the role of DNMT1 in bladder cancer. In the present study, we sought to evaluate the potential role of miRNAs regulated by DNMT1 and the mechanisms responsible for their action in bladder cancer. Here we demonstrated that miR-424 was transcriptionally regulated by DNMT1 activity in bladder cancer cells. Underexpressed miR-424 was noted in bladder cancer specimens compared with adjacent non-malignant specimens and was linked with the prognosis of bladder cancer. Attenuation of the epithelial-to-mesenchymal transition (EMT) and inhibition of EGFR/p-AKT signaling may be the mechanisms underlying the tumor-suppressive role of miR-424 in bladder cancer.

## RESULTS

### DNMT1 regulates the expression of miR-424

**Figure 1:**
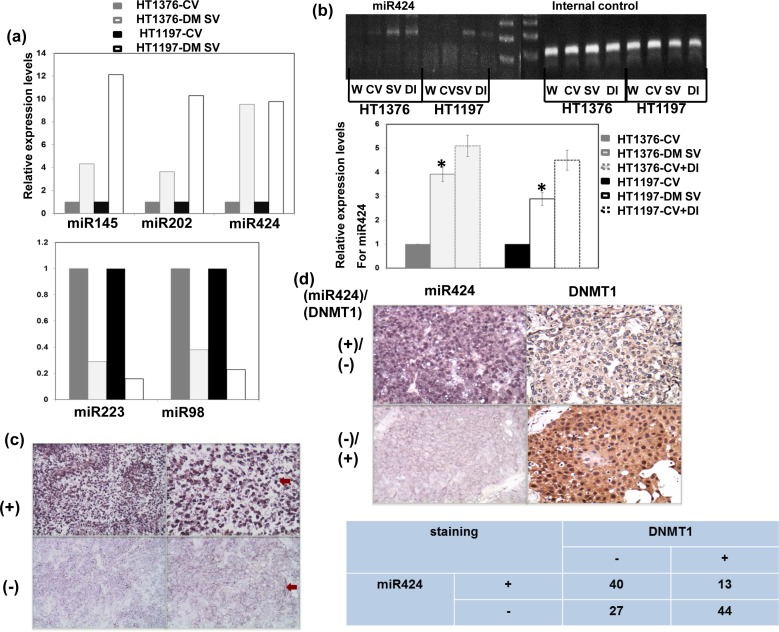
DNMT1 inhibits transcription of miR424 in bladder cancer cells **a.** The changes of miR-424, 145, 202, 223 and 98 levels induced by DNMT1 silencing vector evaluated by Taqman array analysis. Fold change of these microRNAs are expressed relative to cells with control vectors *in vitro*. **b.** Levels of miR-424 with or without DNMT1 silencing vectors or DNMT inhibitors in HT1197 and HT1376 cancer cells examined by real-time RT- PCR (W, wild type; CV, cells transfected with control vectors; SV, cells with DNMT1 silencing vectors; DI, cell treated with DNMT inhibitors). The Y axis shows the ratio of miR424 relative to the respective cells with control vectors. **c.** ISH for miR424 on human bladder cancer specimens. Representative slides demonstrate that tumor cells showed miR424 positive staining (upper row) and negative staining (lower row). Magnification x100 (left panel) and x 200 (right panel). **d.** MiR424 levels were negatively correlated with DNMT1 expression in human bladder cancer specimens (*P* < 0.05). Representative slides of two selected tumor specimens (upper and lower row) demonstrating staining for both miR424 and DNMT1 are shown.

We postulated that alterations in miRNA expression could represent a mechanism responsible for the role of DNMT1 in bladder cancer. To explore this possibility, we used a microRNA Taqman array to analyze the role of DNMT1 in the expression of miRNA in bladder cancer. As shown in Figure 1a, we found that the miR-424, -145 and -202 levels were obviously increased in association with decreased miR-223 and -98 levels in bladder cancer cells with the DNMT1-silencing vector compared with those with control vectors *in vitro*. Next, we performed real-time RT-PCR to quantify the expression levels of miR-424, 98, 145, 223 and 202 in bladder cancers with or without DNMT1 inhibition. The level of miR-424 was more significantly increased in bladder cancer cells with DNMT1-silencing vectors, compared to the others (Figure 1b). The expression of miR-424 and its link to DNMT1 were further evaluated using clinical samples. The ISH data for TMA slides from 124 patients with bladder TCC demonstrated that 53 (43%) showed positive miR-424 immunoreactivity in bladder cancer tissues (Figure 1c). Additionally, the IHC analysis indicated positive staining for DNMT1 in 46% of the bladder cancer tissues, and there was a negative correlation between the expression levels of miR-424 and DNMT1 (Figure 1d). Accordingly, DNMT1 may play a role in the regulation of miR-424 expression in bladder cancer.

### Role of miR-424 in tumor growth and related mechanisms

**Figure 2:**
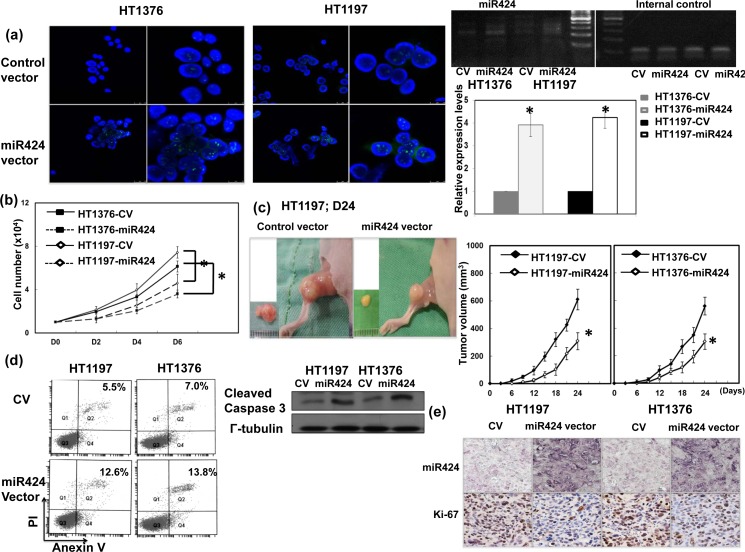
Role of miR424 in tumor cell growth **a.** The level of miR424 in HT1197 and HT1376 transfected with control vectors or miR424 expression vectors demonstrated by FISH and real time RT-PCR. The results are shown by representative slides (DAPI, blue; miR424, green; left panel in LPF, right panel in HPF) (CV, cells transfected with control vectors; miR424, cells with miR424 expression vectors). Levels of miR424 were significantly increased by miR424 expression vector compared to control vector. *, *p* < 0.05. **b.** Effect of miR424 expression vectors on the proliferation rate of HT1197 and HT1376 cancer cells. The same number of cells (10^4^) were plated in each plate on day 0 and allowed to grow in their respective cultures. We counted the number of viable cells after incubation for 2, 4 and 6 days. The Y axis represents the viable cell number. Data points represent the means ± SEMs. *, *p* < 0.05. **c.** Effect of miR424 on tumor xenograft tumor growth. Representative images and quantitation are shown. Data points represent the means ± SEMs. *, *p* < 0.05. **d.** Effect of miR424 expression vector on cell death examined by Flow cytometry using Anexin V-PI staining and the level of cleavage caspase 3 by Western blotting analysis in cells with miR424 expression vectors or control vectors. **e.** Proliferation ratio of xenoraft tumors was evaluated by immunohistochemical staining with antibody to Ki-67 combined with ISH for miR424.

To investigate whether alterations in miR-424 expression play a role in bladder tumor growth, HT1197 and HT1376 bladder cancer cells were transfected with a miR-424 expression vector. As shown in Figure 2a, the miR-424 expression vector significantly increased miR-424 expression in both cell lines. By the counts of viable cells over a period of six days (Figure 2b), the miR-424 expression vector significantly attenuated the proliferation rates of bladder cancer cells. Furthermore, Figure 2c obtained using xenograft tumors demonstrates that increases in miR-424 resulted in slower tumor growth. We further evaluated the changes in cell death and apoptosis-related proteins *in vitro*. Figure 2d and suppl. Figure 1 reveal that the miR-424 expression vectors clearly increased cell death, as determined through flow cytometry and immunodetection of cleaved caspase 3. The IHC staining also demonstrated that the miR-424 expression vector resulted in a significantly decreased Ki-67 proliferation index in xenograft bladder tumors (Figure 2e).

### Role of miR-424 in tumor invasion and epithelial-mesenchymal transition (EMT)

**Figure 3:**
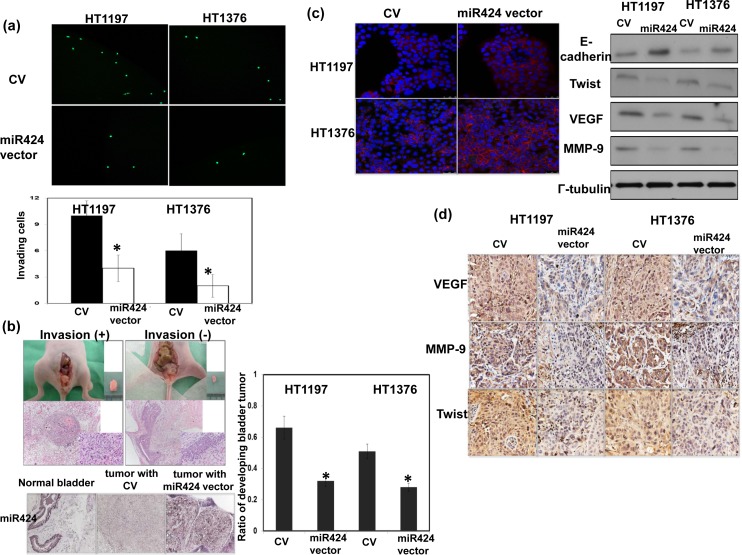
Role of miR424 in tumor invasiveness **a.** Effect of miR424 on the invasive ability of bladder cancer cells. The plates were photographed at indicated time. The Y-axis represents the number of invading cells counted as described in the Materials and Methods. Data points represent the means ± SEMs. *, *P* < 0.05. (CV, cells transfected with control vectors; miR424, cells with miR424 expression vectors). **b.** The invasive capacity of bladder cancer cells was evaluated using murine orthotopic tumor implantation. The representative slides and quantitative data are shown. The y-axis represents the ratio of mice presenting intravesicular tumors normalized to that received orthotopic tumor implantation. Data points represent the means ± SEMs. *, *P <* 0.05. **c.** IF and Western blot analyses demonstrated the effects of miR424 expression vectors on EMT-related proteins in HT1197 and HT1376 cells. Representative micrographs are shown (DAPI, blue; E-cadherin, Red). **d.** Changes in VEGF, MMP-9, and Twist expression in tumor xenografts were evaluated by IHC staining. The results from representative slides are shown.

As demonstrated through the invasion assay, miR-424 expression vector attenuates the invasiveness of bladder cancer cells (Figure 3a). An orthotopic tumor model has been reported to increase the invasive and metastatic potential by tumor cell lines injection into their tissue of origin compared with subcutaneous tumor [16, 17]. Therefore, an orthotopic tumor implantation technique was used to examine the effect of the miR-424 expression vector on invasion ability *in vivo*. As demonstrated in Figure 3b, the miR-424 expression vector decreased the rate of tumor implantation in the bladder and was associated with a smaller tumor size. The EMT is a key event in the invasion process [18], and we determined whether it is the underlying mechanism responsible for the effects of miR-424 on bladder cancer. Figure 3c showed that the miR-424 expression vector increased the epithelial characteristics of bladder cancer cells, as determined by changes in the expression of E-cadherin and twist. Furthermore, the EMT may induce a number of invasion-related factors, including VEGF and MMP-9. As determined through IHC analysis, the miR-424 expression vector resulted in lower expression levels of VEGF and MMP-9 *in vivo* (Figure 3d).

### Expression of miR-424 in patients with bladder cancers

**Figure 4:**
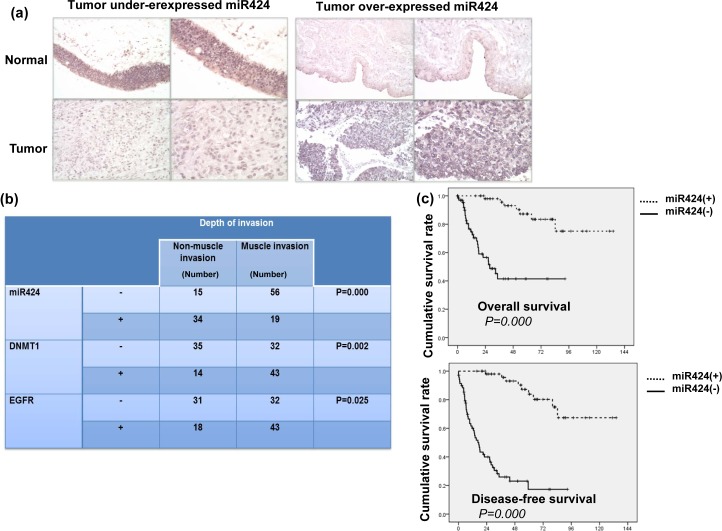
Levels of miR424 on clinical outcome for bladder cancer **a.** Representative slides of ISH for miR424 are shown by images using IPP (Upper row, adjacent non-malignant epithelium; lower row, bladder cancer tissue). Representative slides of two selected tumor specimens (left and right panels) with underexpressed and overexpressed miR424 in cancer tissues compared to adjacent non-malignant epithelium are shown. **b.** The correlation between the depth of tumor invasion and the expression levels of miR4242, DNMT1 and EGFR in clinical samples. The depth of invasion for each tissue specimen was evaluated by a pathologist, and muscle invasion was diagnosed when the cancer has grown through the connective tissue into the muscle layer. **c.** A survival difference was demonstrated in accordance with positive staining of miR424. The Kaplan-Meier overall survival curves showed that patients with lower levels of miR424 expression had shorter survival periods.

The ISH data for the TMA slides contained bladder TCC and adjacent non-malignant epithelium specimens from 45 patients demonstrate that 37 (82%) have lower expression of miR-424 in tumor tissues compared with adjacent non-malignant epithelial tissues (Figure 4a). Of the 124 bladder cancer tissues, 53 (43%) showed positive miR-424 immunoreactivity in bladder cancer tissues. The clinical data revealed a negative correlation between the tumor invasion depth (muscle invasion) and miR-424 expression (Figure 4b). Positive staining for miR-424 was evident in 69% (34/49) of the non-muscle-invasive bladder cancer tissues but only in 25% (19/75) of the T2-T4 bladder cancer tissues (*P* = 0.000). In contrast, 29% (14/49) of the non-muscle-invasive bladder cancer and 57% (43/75) of the muscle-invasive bladder cancer specimens overexpressed DNMT1. Accordingly, our findings suggest that the downregulation of miR-424 that is associated with increased DNMT1 expression significantly correlated with the clinical stage. Regarding the clinical outcome, the staining of miR-424 was significantly linked to a lower recurrence rate and a longer survival in patients after treatment (Table 1 and Figure 4c). As determined in the univariate analysis, positive staining for DNMT1, underexpressed miR-424, high-grade pathology, higher clinical stage (muscle invasion, LN involvement and distant metastasis) and disease failure after treatment were significant predictors for shorter survival. Furthermore, the expression of miR-424 retained predictive power concerning disease-free survival in a multivariate analysis (Table 1b). The findings strongly underscore the contribution of miR-424 to the prognosis in bladder cancer.

**Table 1a:** Baseline characteristics of patients

	No. of patients		*p* value
	miR424 (−)	miR424 (+)	
**Age**			0.29
Median	77	71	
Range	46.5-89.5	44-90	
**Gender**			0.97
Male	44	33	
Female	27	20	
**Depth of invasion**			0.000*
Muscle invasion (−)	15	34	
Muscle invasion (+)	56	19	
**Pathologic grade**			0.000*
Low-intermediate	12	32	
High	59	21	
**LN involvement**			0.006*
Negative	50	48	
Positive	21	5	
**Distant metastasis**			0.35*
Negative	60	51	
Positive	11	2	
**Loco-regional failure**			
Negative	48	45	0.028*
Positive	23	8	
**DNMT1**			0.000*
Negative	27	40	
Positive	44	13	

**Table 1b:** Multivariate analysis to determine molecular markers associated with prognosis (DFS) of patients

Variables	Odd ratios	95% confidence interval	p
DNMT1 staining	2.308	1.222-4.358	0.010
miR424 expression	0.152	0.066-0.350	0.000
Muscle invasion	4.023	1.303-12.428	0.016
Pathology grade	1.764	0.572-5.439	0.323
Pelvic LN involvement	2.232	0.474-10.498	0.310
Distant metastasis	2.865	1.197-6.858	0.018
Loco-regional failure	1.131	0.519-2.469	0.675

### EGFR signaling underlying the role of miR-424 in bladder cancer

**Figure 5:**
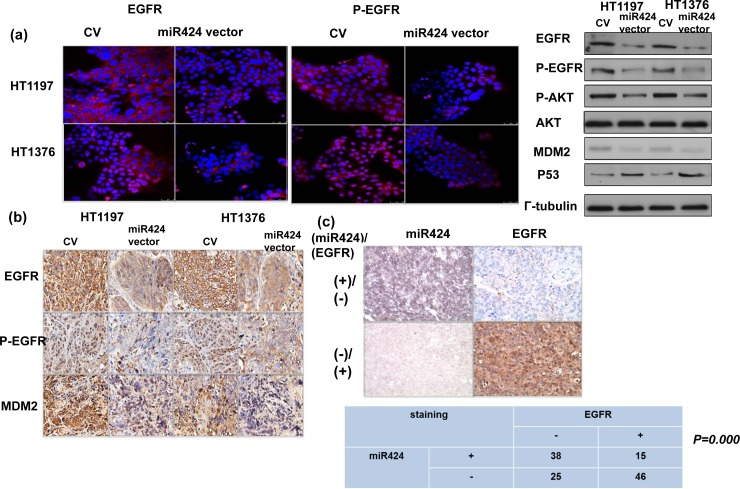

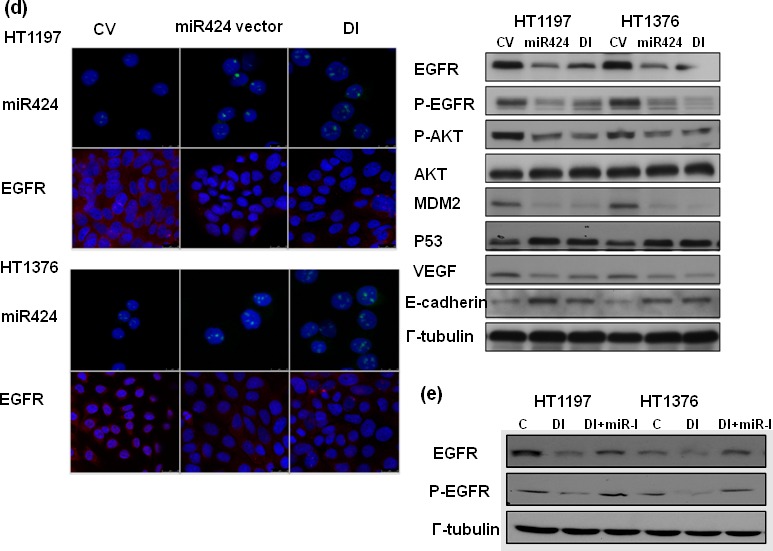
MiR424 linked with EGFR- AKT signaling **a.** Effect of miR424 on the level of EGFR and p-EGFR was examined by immunofluorescence and EGFR-AKT signaling by Western Blotting. The results are shown by representative slides. (Left panel: red, EGFR; Right panel: red, p-EGFR). **b.** Changes in EGFR, p-EGFR, and MDM2 expression in tumor xenografts were evaluated by IHC staining. The results from representative slides are shown. **c.** MiR424 levels were negatively correlated with EGFR expression in human bladder cancer specimens. Representative slides of two selected tumor specimens (upper and lower row) demonstrating staining for both miR424 and EGFR are shown. **d.** Effects of miR424 and DNMT inhibitor on EGFR-AKT signaling were examined by immunofluorescence and Western Blotting analysis. **e.** Effects of miR424 inhibitor combined with DNMT inhibitor on EGFR expression was examined by Western Blotting analysis. (DI, cell treated with DNMT inhibitor; miR-I, cell transfected with miR424-inhibitor).

Many urothelial cancer cell lines, including HT1376 and HT1197, overexpress EGFR [19, 20]. Furthermore, the molecular events underlying the EMT are complex and may play a role in the activation of PI3K signaling [21]. The databases (PicTar and TargetScan) identified EGFR as a predicted target for miR-424. Accordingly, the levels of EGFR and downstream major mediators were investigated to determine whether alterations in EGFR-AKT are involved in the role of miR-424 in bladder cancer. The *in vitro* data in Figure 5a demonstrated that the miR-424 expression vector decreased the levels of EGFR and the major downstream mediators, including p-EGFR and p-AKT. The IHC data using xenograft tumors (Figure 5b) confirmed the *in vitro* findings. Moreover, a significant negative correlation was observed in the clinical specimens between EGFR and miR-424 (Figure 5c). We previously reported that the EGFR-PI3K-AKT pathway may be the mechanism responsible for the aggressive tumor behavior in DNMT1-positive bladder cancer. Therefore, the effect of DNMT1 inhibition on EGFR signaling was evaluated in comparison with that induced by miR-424 *in vitro*. The decreased EGFR signaling induced by the DNMT inhibitor was similar to that induced by miR-424 expression vectors (Figure 5d). Furthermore, Figure 5e & Suppl. Figure 2 demonstrated that the change of EGFR induced by DNMT inhibitor could be reversed by the inhibition of miR424. Accordingly, we suggested that the downregulation of miR-424 mediates the activation of EGFR signaling in DNMT1-positive bladder cancer.

## DISCUSSION

Although alterations in miRNA expression are thought to play an important role in carcinogenesis [4, 22, 23], the mechanisms underlying miRNA dysregulation in bladder cancer are not yet fully understood. DNA methylation has been reported to contribute to the epigenetic silencing of tumor suppressor miRNAs by CpG island hypermethylation [24, 25]. A switch to accumulating DNA hypermethylation is caused by the overexpression of DNMTs [26]. DNMT1 has been reported to regulate the expression of miRNAs in some cancers [8, 27], and participate in multistage urothelial carcinogenesis [28, 29]. A mechanistic understanding of the role of DNA methylation and microRNAs in carcinogenesis may lead to the identification of promising therapeutic avenues for the treatment of cancer. Therefore, the link between DNMT1 and the expression of miRNAs in bladder cancer was evaluated in the present study. We showed that the expression of miR-424 was markedly increased after blocking DNMT1 with silencing vectors or a DNMT inhibitor *in vitro* using microRNA Taqman array and real-time RT PCR assays. Furthermore, the analysis of clinical specimens from bladder TCC patients showed that 53 (43%) had positive miR-424 immunoreactivity in bladder cancer tissues and there was a negative correlation between the expression levels of miR-424 and DNMT1. Accordingly, we suggested that DNMT1 activity is critical for the downregulation of miR-424 expression in bladder cancer.

It has been shown that miR-424 controls many crucial biological activities, including cellular differentiation and proliferation, cell-cycle progression and angiogenesis, all of which are often perturbed in malignancies [30-32]. Aberrant miR-424 expression has been observed in some other cancer types [33-35]. Previous studies have shown that miR-424 may act as a potential tumor suppressor miRNA [33-37]. To examine the role of miRNA 424 in bladder cancer, we stably transfected HT1376 and HT1197 bladder cancer cells with miR-424 expression vectors or control vectors. The data obtained from cellular and animal experiments revealed that the expression of miR-424 was inversely correlated with the tumor growth rate. As determined through protein analysis, increased levels of apoptotic cells *in vitro* and a decreased proliferation index *in vivo* were noted in bladder cancer cells with miR-424 expression vectors. Additionally, we found that the miR-424 expression vector significantly attenuated the invasive ability of cells, as determined through cellular invasion assays and mouse orthotopic models. The molecular and phenotypic changes involved in the EMT appear to be functionally relevant to the invasive characteristics of epithelial tumors, including bladder cancer [18, 38]. At the molecular level, the EMT is characterized by a loss of E-cadherin, a hallmark of EMT, and increased expression of invasion-related factors [39]. We demonstrated that increasing miR-424 expression abolished the expression of twist, VEGF and MMP-9, which are associated with increased E-cadherin expression. Based on these findings, the attenuation of the EMT may be responsible for the reduced invasiveness of miR-424-overexpressing bladder cancer cells.

The identification and selection of molecular targets are important in cancer therapy. The cell line data presented herein highlight the potential pivotal roles of miR-424 in both the development and progression of bladder cancer. We further evaluated the predictive role of miRNA 424 in patients with bladder cancer. Our ISH data obtained from clinical samples reveal that miR-424 is expressed at lower levels in bladder cancer specimens compared with adjacent non-malignant specimens. The predictive powers of miRNA 424 and DNMT1 were further examined in terms of the clinical outcomes of bladder cancer. The data demonstrated a negative correlation between DNMT1-positive samples and those expressing miR-424. The underexpression of miR-424 associated with a positive staining for DNMT1 was preferentially associated with muscle-invasive bladder TCC relative to non-muscle-invasive disease. Moreover, we uncovered a parallel association of low miR-424 expression with poor prognostic factors, such as advanced clinical stage, node metastasis, and high pathological grade, suggesting that miR-424 may play a suppressive role in the progression of bladder cancer. Our data also showed that an enhanced expression of miR-424 is a significant predictor of progression-free survival in bladder cancer. Consistent with the clinical data, the restoration of miR-424 expression in bladder cancer cells markedly affects the cellular biological behaviors, including suppressed proliferation, enhanced apoptosis and inhibited migration and invasion. Thus, our *in vitro* and *in vivo* findings together suggest that miR-424 functions as a tumor suppressor and that the inhibition of miR-424 expression contributes to the progression of bladder cancer by inducing or promoting malignant cellular behaviors and may even predict poor patient prognosis.

**Figure 6:**
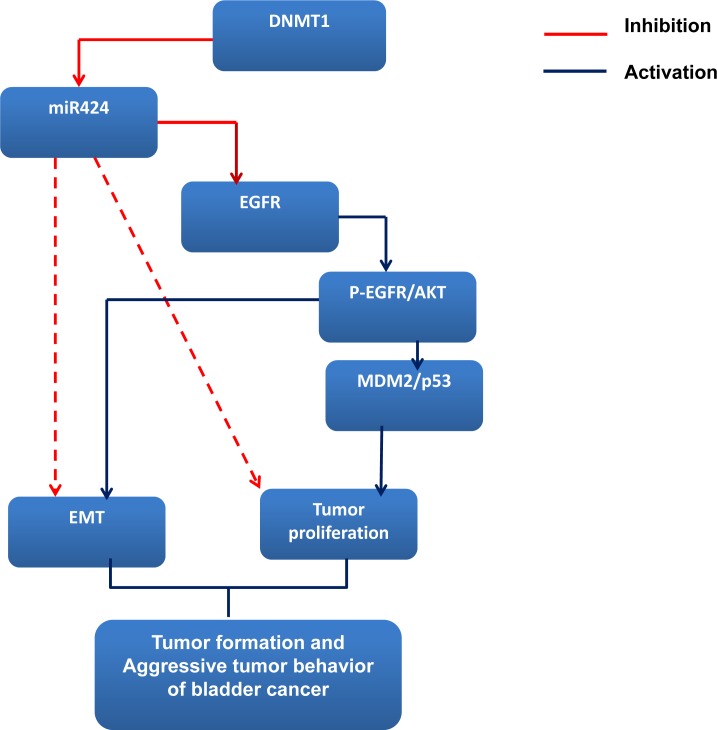
miR424 signaling pathway in bladder cancer

Although the suppressive role of miR-424 in the progression of bladder cancer was explored, a lack of knowledge regarding the miRNA targets hampers a full understanding of the biological functions of aberrantly expressed miRNA. miR-424 is predicted to target many mRNAs (TargetScan and PicTar), including EGFR, Twist and DNA damage-repair genes. Many urothelial cancer cell lines, including HT1376 and HT1197, overexpress EGFR [19, 20]. EGFR is widely reported to be a molecular marker expressed in bladder cancer, and upregulated EGFR signaling is known to be a poor prognostic predictor [40, 41]. The EGFR-mediated PI3K-AKT pathway significantly influences a wide range of cellular processes including cellular growth, resistance to apoptosis and transition of the EMT [18, 21]. Furthermore, EGFR is one of the major cancer-related signaling molecules to be controlled by miRNAs [6, 42]. Our previous data indicated that the EGFR-AKT pathway mediated the aggressive tumor behavior in DNMT1-positive bladder cancer [14]. Accordingly, we evaluated the status of EGFR in terms of the effects induced by miR-424. The data *in vitro* and *in vivo* indicated that the miR-424 expression vector attenuated EGFR and p-EGFR expression associated with decreases in phosphorylated AKT. Moreover, the decrease in EGFR and p-AKT induced by blocking DNMT1 was similar to that induced by miR-424. Through protein analysis, we also found that the expression of MDM2, an oncogene downstream of AKT phosphorylation, was decreased in association with increased p53 in cancer cells with miR-424 expression vectors. The IHC data obtained from clinical samples revealed that miR-424 expression is inversely correlated with EGFR staining, confirming the *in vitro* and *in vivo* findings. Accordingly, it is likely that the inhibition of the EGFR-mediated PI3K-AKT pathway plays an important role in the transmission of the miR-424 signal to downstream targets that regulate cell growth, EMT and invasiveness. The data obtained from the present study reveal that increased miR-424 production plays an important tumor-suppressive role in bladder cancer. We outlined the main signaling pathways that are thought to link DNMT1 and miR-424 to bladder cancer (Figure 6).

Taken together, our findings show that miR-424 is downregulated in bladder cancer tissues and correlated with progression of the disease and that miR-424 regulates multiple cellular biological behaviors, such as retarding growth, inducing apoptosis, and reducing invasion, by directly targeting EGFR in bladder cancer cells. MiRNAs that play an important role in tumor formation and/or metastasis can serve as targets for therapeutic intervention, such as miRNA replacement therapy. Therefore, these results highlight a potential role for miR-424 as a molecular predictor and therapeutic target in bladder urothelial carcinoma.

## MATERIALS AND METHODS

### Tissue specimens and characteristics of patients

The Institutional Review Board of our hospital approved the present study. Informed written consent for the acquisition and storage of medical information and tissue specimens was obtained from all patients. A total of 124 patients with bladder TCC, including 75 with muscle-invasive tumors (the cancer has grown through the connective tissue into the muscle layer) and 49 with non-muscle-invasive tumors (tumors are confined to epithelium or subepithelial connective tissue), were enrolled in the study. The characteristics of the enrolled patients are listed in Table 1a. Specimens retrospectively collected from these 124 patients were prepared into tissue microarray (TMA) blocks by AutoTiss 1000 (Ever BioTechnology, Canada) for immunochemical analysis. Moreover, the TMA block contained bladder TCCs and adjacent non-malignant epithelium specimens from 45 patients. In cases in which blocks were available, the hematoxylin-and-eosin-stained slides were reevaluated by a pathologist to assess the quality of the TMA slides. Data concerning the initial diagnosis, staging, pathological factors, recurrence and survival were collected.

### Immunohistochemical staining (IHC) and in situ hybridization (ISH)

Formalin-fixed, paraffin-embedded tissues were cut into 4-μm sections, mounted on slides, deparaffinized with xylene and dehydrated using a graded ethanol series. Antigen retrieval using citric acid (pH 6.0) at 97°C for 30 min was followed by treatment with 3% hydrogen peroxide. The slides were incubated overnight at 4°C with antibodies against specific proteins. After three washes in phosphate-buffered saline (PBS), the sections were incubated with biotinylated secondary antibody for 10 min, stained with peroxidase-avidin and washed again in PBS, and then 3-amino-9-ethylcarbazole solution was added. DNMT1 immunoreactivity was considered positive if the cells showed positive nuclear staining, regardless of cytoplasmic staining. The sections were counterstained with hematoxylin. The IRS was calculated by multiplying the staining intensity by the percentage of positively stained cells. The criterion for positive staining was an IRS score of at least 2. The expression of miR-424 in bladder cancer specimens was determined using a biotin-labeled probe (Exiquon, Woburn, MA, USA) and an *in situ* hybridization detection kit (Dako, Glostrup, Denmark) according to the manufacturer's instruction.

### MicroRNA expression profiling

The total RNA from the cells stably transfected with DNMT1-silencing vectors or control vectors was extracted with Trizol reagent solution (Ambion) to quantify microRNAs. Expression of miRNAs was analyzed using a TaqMan MicroRNA array (Life Technologies). Briefly, 1 mg of total RNA was reverse-transcribed using a Megaplex Pools kit (Applied Biosystems, Foster City, CA, USA), after which miRNAs were amplified and detected using polymerase chain reaction (PCR) with specific primers and TaqMan probes. U6 was used as an endogenous control. The gene expression is expressed as the ratio of expression relative to that in the control.

### Quantitative real-time polymerase chain reaction of miRNA

The total RNA from the cells transfected with DNMT1-silencing vectors or control vectors with or without 10 μM DNMT inhibitor for 24 h was extracted with Trizol reagent solution (Ambion) to quantify microRNAs. Expression of selected miRNAs was analyzed using a TaqMan Human MicroRNA Assay kit (Applied Biosystems, Foster City, CA). Briefly, 5 ng of total RNA were reverse-transcribed using specific stem-loop real-time primers, after which they were amplified and detected using PCR with specific primers and TaqMan probes. U6 snRNA (RNU6B, Life Technologies) served as an endogenous control.

### Cell culture and reagents

Four human bladder cancer cell lines, namely HT1376 and HT1197, were obtained from the American Type Culture Collection (ATCC). We maintained the bladder cancer cell lines in Dulbecco's modified Eagle's medium supplemented with 10% fetal bovine serum. The DNMT inhibitor 5-aza-2′-deoxycytidine (5-AZDC) and miR424 inhibitor were purchased from Sigma (St. Louis, MO, USA) and Life Technologies, respectively. The DNMT1-GFP silencing vector and GFP-control vector were purchased from InvivoGen, as previously described [14]. The miR-424 expression vector (human miR-424 constructs in LentiIII vector) and control vector were obtained from Applied Biological Materials (ABM) Inc. (Richmond, Canada). Stable miR-424-expressing cancer cells were generated by transfecting bladder cancer cells with the miR-424 expression vector and selected by culturing in medium containing puromycin for four weeks.

### Ectopic and orthotopic tumor xenograft model

This study was performed in accordance with the recommendations in the Guide for the Care and Use of Laboratory Animals as promulgated by the Institutes of Laboratory Animal Resources, National Research Council, USA. The protocol was approved by the Committee on the Ethics of Animal Experiments at our Hospital. Eight-week-old female athymic nude mice were used as the xenograft tumor implantation model. In the ectopic tumor implantation model, 1 × 10^7^ tumor cells were subcutaneously implanted by injection into the dorsal gluteal region (five animals/group). The tumor size was measured every three days after implantation (day 0). The tumor volume was calculated assuming an ellipsoid shape. In the orthotopic tumor implantation model, we performed intravesical instillation of cancer cells as previously described (five animals/group) [14]. The extent of orthotopic tumor invasion was measured after implantation at the indicated times.

### Cell invasion assay

The invasiveness of cells was determined using a Cell Invasion Assay (Trevigen, Gaithersburg, MD). After incubation for 24 h, the number of cells in the bottom chamber was determined by measuring the fluorescent anion calcein, which was released from intracellular calcein acetoxymethylester.

### Immunofluorescence (IF) staining

The cells were seeded onto glass coverslips at a density of 5 × 10^4^ cells/ml in six-well plates for IF staining with or without treatment. After treatment for the specified times, the cells were fixed with 2% paraformaldehyde for 5 min and washed in phosphate buffered saline (PBS) with Tween-20 (PBST). The slides were incubated for 1 h at room temperature with antibodies against E-cadherin and cleaved caspase 3 and then with a FITC-conjugated secondary antibody for 1 h and counterstained with DAPI to visualize the nuclei. The expression of miR-424 in bladder cancer cells *in vitro* was determined using a fluorescein-labeled probe (Exiquon, Woburn, MA, USA) according to the manufacturer's instructions.

### Statistical analysis

The survival probabilities were analyzed using the Kaplan-Meier method. Survival was calculated from the date at which treatment was started until the date of death or the most recent follow-up. The significance of the differences between groups was assessed using the log-rank test. The data are presented as the means ± standard error of the mean (SEM). A probability level of *p* < 0.05 was adopted throughout the study to determine significance unless otherwise stated.

## SUPPLEMENTARY MATERIAL FIGURES


