# Supplementary material for: DNMT1-dependent suppression of microRNA424 regulates tumor progression in human bladder cancer
Source: Oncotarget. 2015 Jun 10;6(27):24119–31. doi: 10.18632/oncotarget.4431 (PMC4695174; doi:10.18632/oncotarget.4431)
Supplement: Supplementary file 1 [file oncotarget-06-24119-s001.pdf]

DNMT1-dependent suppression of microRNA424 regulates tumor progression in human bladder cancer

Supplementary Material

Supplementary Figure 1

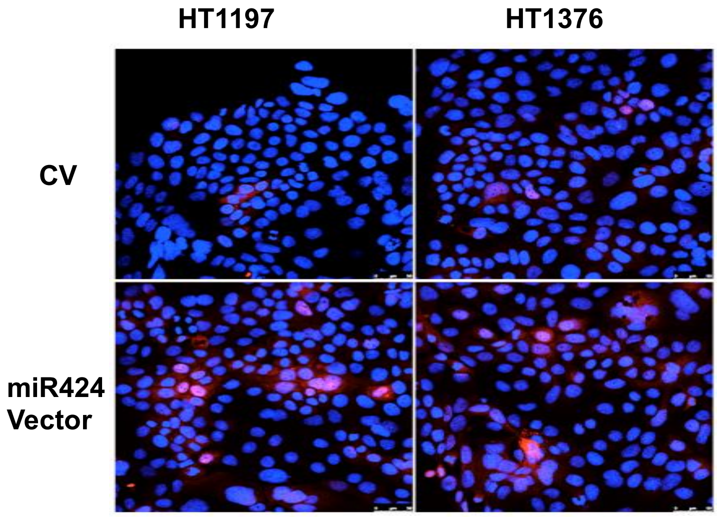

Figure 1: Role of miR424 in tumor cell death

Effect of miR424 expression vectors on cell death examined by IF in cells with miR424 expression vectors or control vectors (DAPI, blue; cleaved caspase 3, red)

Supplementary Figure 2

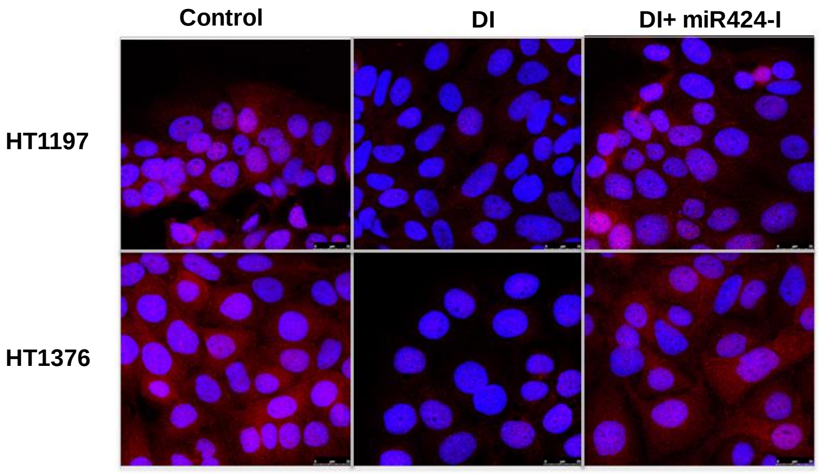

Figure 2: MiR424 linked with EGFR expression

Effects of miR424 inhibitor combined with DNMT inhibitor on EGFR expression was examined by immunofluorescence analysis. (DI, cell treated with DNMT inhibitor; miR-I, cell transfected with miR424-inhibitor)
